# Family health strategy and equity in prenatal care: a population based cross-sectional study in Minas Gerais, Brazil

**DOI:** 10.1186/s12939-016-0503-9

**Published:** 2017-01-21

**Authors:** Mônica Viegas Andrade, Kenya Valéria Micaela de Souza Noronha, Allan Claudius Queiroz Barbosa, Michelle Nepomuceno Souza, Júlia Almeida Calazans, Lucas Resende de Carvalho, Thiago Augusto Hernandes Rocha, Núbia Cristina Silva

**Affiliations:** 1 CEDEPLAR, Federal University of Minas Gerais - UFMG, Av. Antônio Carlos 6627, sala 3006, Belo Horizonte, MG 31270-901 Brazil; 2 CEPEAD, Federal University of Minas Gerais - UFMG, Av. Antônio Carlos 6627, sala 3040, Belo Horizonte, MG 31270-901 Brazil; 3 Department of Global Health and Population, Harvard T.H. Chan School of Public Health, Boston, MA USA

**Keywords:** Antenatal care, Prenatal care, Primary health care, Family health strategy, Health equity, Brazil

## Abstract

**Background:**

Prenatal care coverage is still not universal or adequately provided in many low and middle income countries. One of the main barriers regards the presence of socioeconomic inequalities in prenatal care utilization. In Brazil, prenatal care is supplied for the entire population at the community level as part of the Family Health Strategy (FHS), which is the main source of primary care provided by the public health system. Brazil has some of the greatest income inequalities in the world, and little research has been conducted to investigate prenatal care utilization of FHS across socioeconomic groups. This paper addresses this gap investigating the socioeconomic and regional differences in the utilization of prenatal care supplied by the FHS in the state of Minas Gerais, Brazil.

**Methods:**

Data comes from a probabilistic household survey carried out in 2012 representative of the population living in urban areas in the state of Minas Gerais. The sample size comprises 1,420 women aged between 13 and 45 years old who had completed a pregnancy with a live born in the last five years prior to the survey. The outcome variables are received prenatal care, number of antenatal visits, late prenatal care, antenatal tests, tetanus immunization and low birthweight. A descriptive analysis and logistic models were estimated for the outcome variables.

**Results:**

The coverage of prenatal care is almost universal in catchment urban areas of FHT of Minas Gerais state including both antenatal visits and diagnostic procedures. Due to this high level of coverage, socioeconomic inequalities were not observed. FHS supplied care for around 80% of the women without private insurance and 90% for women belonging to lower socioeconomic classes. Women belonging to lower socioeconomic classes were at least five times more likely to receive antenatal visits and any of the antenatal tests by the FHS compared to those belonging to the highest classes. Moreover, FHS was effective in reducing low birthweight. Women who had prenatal care through FHS were 40% less likely to have a child with low birthweight.

**Conclusion:**

This paper presents strong evidence that FHS promotes equity in antenatal care in Minas Gerais, Brazil.

**Electronic supplementary material:**

The online version of this article (doi:10.1186/s12939-016-0503-9) contains supplementary material, which is available to authorized users.

## Background

Prenatal care encompasses a wide spectrum of clinical procedures and assistance for pregnant women to improve maternal and child health. Not only are the benefits of prenatal care widely recognized in the literature - - but in low and middle income countries, appropriate prenatal interventions are cost-effective [1–7]. Notwithstanding all the evidence of the benefits of prenatal care, (including reduction of neonatal tetanus, reduction of low birthweight and preterm-delivery, and screening and treatment of infectious diseases), its coverage is still not universal or adequately provided in many low and middle income countries [2, 8–13]. Limited prenatal coverage remains one of the main barriers that signifies the presence of socioeconomic inequalities in prenatal care utilization. Socioeconomic (education, income and gender) inequalities matter for access to prenatal care. For instance less educated and lower income women have less information and resources to seek care. Besides, they usually have less confidence in healthcare providers and lower autonomy in households and live in rural/remote areas. In these areas, access to healthcare and transportation facilities is limited since they are in lower supply [9, 14–23].

In Brazil, prenatal care is supplied for the entire population at the community level as part of the Family Health Strategy (FHS), which is the main source of Primary Health Care (PHC) provided by the public health system. FHS has played an important role in the prevention of diseases and the promotion of health awareness since it constantly monitors families through systematic household visits by Family Health Teams (FHT). According to this strategy, families are the focus of public health policies that cover primary care for all population groups, from the newborn to the elderly individuals, irrespective of their health conditions. Family Health Teams (FHT) are mainly composed of family physician, nurse, nursing assistant and at least 4-12 community health agents (CHA) and are based in Health Units. Each FHT is responsible for at most 3,450 peopled living a catchment area [24] . The FHT must be able to detect symptoms of disease and to refer individuals to the needed healthcare unit. In addition, health promotion and disease prevention activities such as encouraging child immunization, antenatal care, and special care to individuals who suffer from diabetes, hypertension or coronary diseases are stimulated. Personal and household hygiene advices are also provided by the CHA in order to prevent and control infectious diseases, especially those caused by vector-borne viruses and bacteria [24, 25]. CHA play a fundamental role as they act as a bridge between the population and Health Units. They are responsible for families’ enrolling and are required to visit each household on a monthly basis in order to identify risk factors and vulnerable conditions. Besides, CHA are required to promote active search of pregnant woman and refer them to receive prenatal care. This protocol contributes to strengthen the bond between health professionals and pregnant women.

Some empirical evidence has already been raised about the importance of FHS showing a robust impact on reducing infant mortality [26–28] and decreasing hospitalizations due to conditions sensitive to primary care [29–31]. Related to antenatal care FHS has contributed to scale up the coverage and to promote an equal access to adequate care [32, 33]. However, Brazil has some of the greatest income inequalities in the world [34]. Additionally little research has been conducted to investigate prenatal care provided by FHS across socioeconomic groups.

This paper addresses this gap by investigating the disparities in the utilization of prenatal care supplied by the FHS in the state of Minas Gerais, Brazil, using data from a population-based study conducted through a household survey. Minas Gerais is the third largest economy in Brazil and has strong socioeconomic disparities. Hence, its regional heterogeneity largely reflects that found in Brazil, making it representative of the country’s socioeconomic and epidemiological profiles [35].

## Methods

**Fig. 1:**
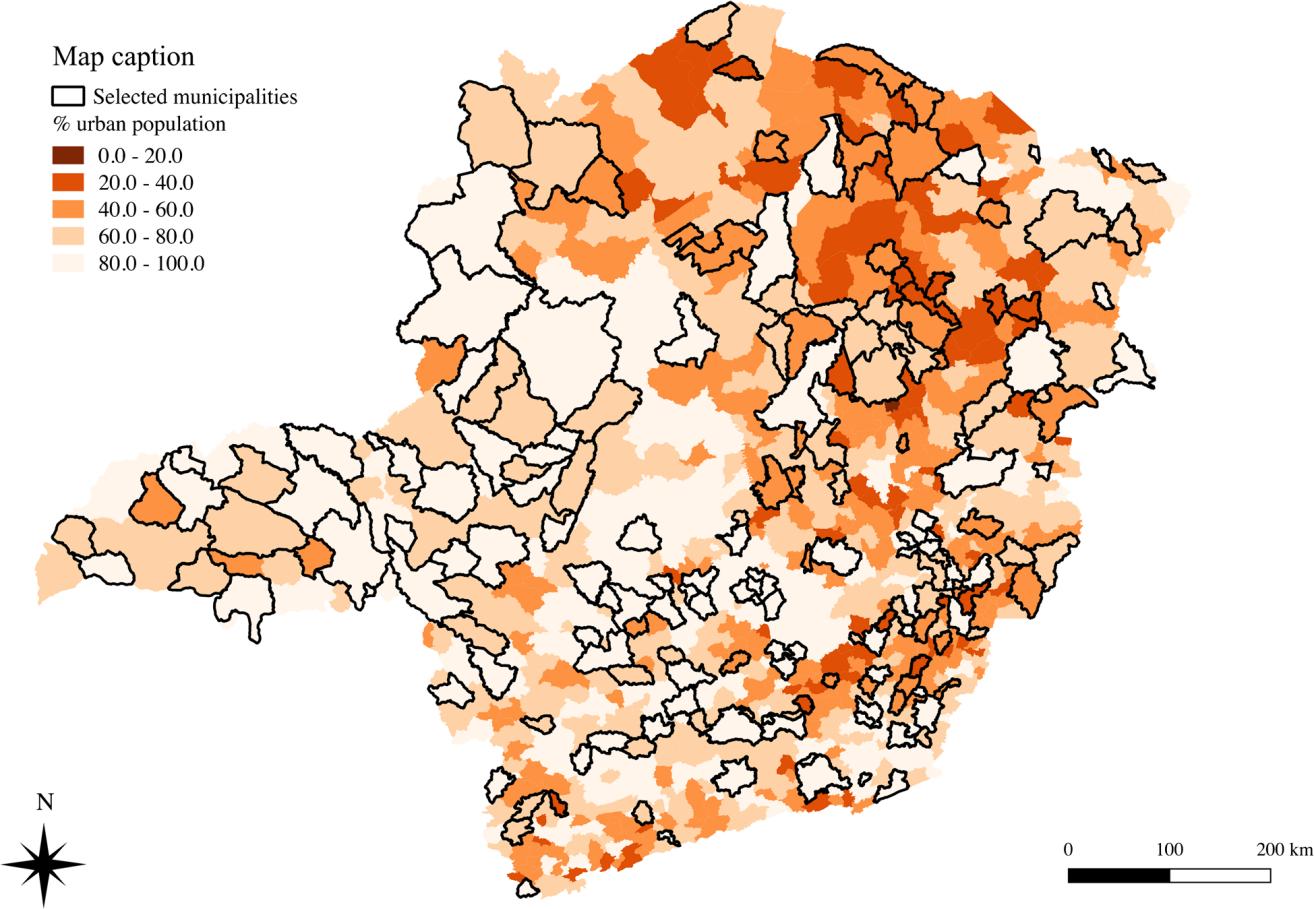
Percentage of population living in urban areas in each Municipality of Minas Gerais state and selected municipalities investigated in the survey

This study is classified as cross sectional-ecological based on primary databases. Data were obtained from a household survey undertaken in Minas Gerais in 2012. This survey investigated healthcare services provided by the Family Health Strategy (FHS). A probabilistic multi-stage sample stratified by the thirteen health macro regions was defined in order to be representative of the population living in urban areas in the state of Minas Gerais [36]. The primary and secondary units sampling were respectively FHT and households. The definition of the number of FHT and households surveyed was based on the total of FHT in Minas Gerais in 2011 considering a margin of error of 5%. Therefore, 208 FHT were investigated in the State and equally distributed among the 13 health macro regions. The selection of municipalities took into account the socioeconomic disparities observed in the state measured by the Municipal Human Development Index (MHDI) and the number of FHT [35]. A total of 173 municipalities were selected and distributed in order to guarantee that 16 FHT were investigated in each health macro region. After the selection of municipalities, households were randomly drawn in geographical catchment areas of each health units. Since not all Health Units had available a map of catchment areas, we used the Official Register of Addresses provided by the Brazilian Institute of Geography and Statistics to identify the households (Fig. 1) located in the geographical area of each health unit, named here as potential catchment areas [37]. To estimate the number of households located in these potential catchment areas, the Health Units were georeferenced using the software ArcGIS and two assumptions were made: 1) each FHT provides PHC on average for 3,450 individuals; and 2) it was considered an average of four persons per household. In that manner, each potential catchment area would include 850 households.

The sample size comprises 1,420 women aged between 13 and 45 years old who had completed a pregnancy term with a live birth in the last five years prior to the survey. These women were selected if their child under five years old were living with their mothers in the same household. Mothers whose children did not survived were not included in the sample. As a result, maternal or infant mortality were not possible to be analyzed. In the case of more than one pregnancy during the five-year-period, only the pregnancy of the youngest surviving child was investigated. This study was approved by the institutional review board of the Federal University of Minas Gerais, protocol # 04200203000-10.

### Variables

The outcome variables for the study were received prenatal care, number of antenatal visits, late prenatal care, antenatal tests and tetanus immunization. Antenatal visits were categorized into less than 6 and 6 or more visits as recommended by the Brazilian Ministry of Health [38, 39]. Late prenatal care was defined as having received the first antenatal visit after three months of pregnancy. The antennal tests included blood, urine, Sexually Transmitted Disease (STD) and toxoplasmosis test. As the tetanus immunization ensures protection during a ten-year period, some women do not take the vaccine during pregnancy since they have already been immunized before. Failure to consider this information may underestimate immunization coverage against tetanus. In this study, the coverage of tetanus vaccine immunization was estimated combining the information regarding immunized mothers during pregnancy with those who were already immunized.

Besides antenatal care components, we also investigated low birthweight using World Health Organization definition: infants born weighting less than 2,500 grams regardless of gestational age [40]. Since the Brazilian Health System is mixed, we also investigated the types of providers that supplied antenatal care that allowed for the role of FHS to be distinguished from that of the other antenatal care providers. Three types of providers were considered: FHS, other public health care facilities and private care (out-of-pocket and health insurance).

The independent variables for the study comprise the socio-demographic characteristics of the pregnant women such as mother’s age during pregnancy, socioeconomic class, mother’s level of education, region of residence, private health insurance coverage and diagnosis of hypertension and diabetes at the moment of the interview. Educational level was defined by an indicator with four categories representing the completed education level achieved by the respondents that were interviewed: 1) less than completed middle school; 2) completed middle school and incomplete high school; 3) completed high school; 4) over high school. Socioeconomic class is a categorical variable defined by the Brazilian Association of Research Companies [41]. This criterion classifies the population according to possession of household goods, number of domestic employees, and the highest educational level in the household. A wealth index was built for each household that was classified into three socioeconomic classes: A/B, C, and D/E. A descriptive analysis and multivariate logistic models were generated based on the outcome variables.

## Results

Table 1 presents the distribution of characteristics of the women that were interviewed. The majority of them belonged to the middle class and had at least completed middle school. Around 25% were covered by private health insurance reflecting the same distribution observed for Brazil [42]. Regarding age, 14% was classified as risky pregnancy since 11% were aged between 10 to 19 years old (adolescent pregnancy) and 3.5% were over 39 years. Only 2% and 13% of the women reported suffering from Diabetes and Hypertension respectively.

**Table 1:** Distribution of women by socioeconomic characteristics and health status, Minas Gerais, 2012

	Number	Percent
Educational level		
Less than completed middle school	251	17.66
Completed middle school and incomplete high school	546	38.46
Completed high school	535	37.65
Over high school	88	6.23
Region of residence		
Central	573	40.32
North	224	15.78
South	189	13.28
Triângulo Mineiro	114	8.05
Zona da Mata	191	13.43
East	130	9.13
Economic class		
A-B	325	22.90
C	753	53.02
D-E	342	24.08
Age Group		
10 to 19	164	11.54
20 to 24	403	28.38
25 to 29	386	27.21
30 to 39	417	29.35
over 40 years	50	3.51
Health insurance		
No coverage	1059	74.60
Coverage	361	25.40
Total	1,420	100.00
Diabetes		
No	1237	97.96
Yes	26	2.04
Total	1,263	100.00
Hypertension		
No	1099	86.87
Yes	166	13.13
Total	1,265	100.00

Note 1: Central = Center + South Center; North = Jequitinhonha + Northwest + North of Minas + Northeast; South = South + West; Triâgulo = North Triângulo + South Triângulo; Zona da Mata = East of South + Southeast; East = East. Note 2: 11.52% of sample has been omitted to diabetes and 11.33% to hypertension

In the potential catchment areas, prenatal coverage was almost universal, evidencing the success of this policy in Minas Gerais. Only 15 (1.06%) women did not have access to antenatal care (results not shown here). FHT supplied care for 68.47% of the total women who received prenatal care and among those without private health insurance, this percentage increased to 79.51% (Table 2). These findings revealed that the FHS is the major provider of prenatal care in Minas Gerais. Additionally, FHS coverage is higher among the lower socioeconomic classes. For example, for women without private health insurance and belonging to the D-E classes about 90% have had prenatal care through FHS while among those belonging to the A and B 68% indicated that they have had prenatal care through FHS.

**Table 2:** Percentage distribution of women who received prenatal care, total and without private health insurance according to economic class (%), Minas Gerais, 2012

Economic Class	Total (*N* = 1405)			Without private health insurance (*N* = 1069)		
	FHS	Other Public Providers	Private Providers	FHS	Other Public Providers	Private Providers
A-B	45.97	3.73	50.31	68.22	4.75	27.03
C	69.11	8.25	22.64	77.29	8.09	14.62
D-E	88.72	7.09	4.18	89.86	7.14	3.00
Total	68.47	6.92	24.61	79.51	7.24	13.25

Table 3 displays the results for the multivariate logistic model that estimated the likelihood of having received prenatal care by the FHS. The results showed that the chance of having received prenatal care by FHS is five times higher among the lower socioeconomic groups (D-E classes) than A-B classes. The results observed for the other socioeconomic indicators such as private health insurance and level of schooling reinforce this pattern of the utilization of antenatal care services: women without private health insurance and with lower educational level are more prone to receive care by FHT. Significant regional differences were observed for women living in the North and Triangulo Mineiro regions, where the odds of receiving prenatal care from FHS were higher than in the Central region.

**Table 3:** Logistic regression for receiving prenatal care provided by the FHS, Minas Gerais, 2012

Variable	Odds ratio	*P*-values
Age	0.97^***^	0.010
Private health insurance	0.22^***^	<0.001
Class C (*ref. Class A and B*)	1.81^***^	0.001
Class D-E	5.09^***^	<0.001
North Region (*ref. Central*)	1.66^**^	0.035
South Region	0.99^NS^	0.969
Triangulo Mineiro Region	4.06^***^	<0.001
Zona da Mata Region	1.04 ^NS^	0.859
East Region	0.77 ^NS^	0.322
Diabetes	0.33^**^	0.020
Hypertension	0.87 ^NS^	0.490
Belo Horizonte (Capital of the State)	2.02^***^	0.003
Completed middle school and incomplete high school (*ref. Less than completed middle school*)	0.75 ^NS^	0.222
Completed high school	0.51^***^	0.003
Over high school	0.66 ^NS^	0.239
Constant	5.11^***^	0.001
Number of observations	1,250	
Adjusted R^2^	0.20	
Log of Likelihood	-638.32	

Note: Statistically significant at 1% margin of error (Sig. level = 99%); ^**^Statistically significant at 5% margin of error (Sig. level = 95%). NS not significant. ^***^Statistically significant at 1% margin of error (Sig. level = 99%)

In Minas Gerais at least 90% of women received more than 6 antenatal care visits independent of the type of provider and individual characteristics (Table 4). Regarding quality of antenatal care, this survey investigated late prenatal care, tetanus immunization and antenatal tests. Table 4 indicates that 10.28% of women received late prenatal care and only 3.49% was not immunized. The high prevalence of late prenatal care seems to be associated with adolescent pregnancy (18.53%) and socioeconomic status (16.25% among D-E classes).

**Table 4:** Prevalence of women who received less than 6 ANC visits (total and by FHS), late prenatal care and were not immunized according to socioeconomic characteristics (%), Minas Gerais, 2012

	Less than 6 ANC visits		Not immunized	Late PNC
	(*N* = 1,392)	(*N* = 980)	(*N* = 1,378)	(*N* = 1,389)
	Total	FHS	Total	Total
Region of residence				
Central	8.91	8.76	3.56	11.33
North	8.98	10.54	3.99	10.67
South	8.75	10.45	5.44	9.93
Triângulo	5.67	5.95	2.60	6.49
Zona da Mata	4.97	2.72	3.45	10.02
East	5.68	5.72	4.91	9.31
Economic Class				
A-B	8.24	10.01	3.48	5.53
C	6.90	6.93	3.60	9.71
D-E	9.43	8.96	5.07	16.25
Age Group at pregnancy				
10 to 19	16.06	15.03	2.99	18.53
20 to 24	9.72	8.69	5.10	12.19
25 to 29	4.70	4.28	2.91	9.07
30 to 39	6.20	7.52	4.40	7.26
Over 40 years	2.56	2.58	1.41	1.61
Education level				
Less than completed middle school	8.44	8.12	3.39	12.27
Completed middle school and incomplete high school	11.65	9.76	5.83	14.88
Completed high school	4.22	5.63	2.27	5.69
Over high school	4.30	8.82	3.43	4.66
Private Health Insurance				
No	8.06	8.23	4.07	11.99
Yes	7.10	6.86	3.45	5.38
FHS prenatal care				
No	-	-	4.58	8.51
Yes	-	-	2.99	11.10
Total	7.81	8.04	3.49	10.28

The results for antenatal tests showed that the coverage was almost 100% for blood test, urine and STD (Additional file 1: Table S1). The main difference was found only for the toxoplasmosis test, which had a lower coverage, less than 90%. Besides, it was noticed that about 50% of each antenatal test were performed by the FHS (Additional file 1: Table S1).

Table 5 shows the chance of having undertaken each prenatal test by FHS. The results show that this probability is inversely associated to socioeconomic status: women belonging to D-E class had at least 5 times more chance to undertake any antenatal test by the FHS compared to those belonging to the A-B classes.

**Table 5:** Logistic regression for receiving prenatal tests by the FHS, Minas Gerais, 2012

Variable	Blood test		Toxoplasmosis		Urine test		STD test	
	Odds Ratio	*P*-values	Odds Ratio	*P*-values	Odds Ratio	*P*-values	Odds Ratio	*P*-values
Age	0.99	0.329^NS^	0.99	0.481^NS^	1.00	0.723^NS^	0.98	0.210^NS^
Private health insurance	0.18	0.000***	0.22	0.000***	0.16	0.000***	0.18	0.000***
Class C (*ref. Class A and B*)	1.80	0.001***	1.68	0.006***	1.90	0.001***	2.01	0.000***
Class D-E	6.19	0.000***	5.96	0.000***	8.15	0.000***	6.40	0.000***
North Region (*ref. Central*)	0.99	0.960^NS^	0.95	0.840^NS^	0.98	0.933^NS^	0.96	0.883^NS^
South Region	0.75	0.234^NS^	0.64	0.060*	0.69	0.128^NS^	0.69	0.126^NS^
Mineiro Triangle Region	6.75	0.000***	6.22	0.000***	6.37	0.000***	6.42	0.000***
Zona da Mata Region	0.71	0.151^NS^	0.90	0.656^NS^	0.75	0.236^NS^	0.74	0.219^NS^
East Region	0.71	0.224^NS^	0.54	0.027**	0.66	0.138^NS^	0.58	0.048**
Diabetes	0.77	0.624^NS^	0.71	0.529^NS^	0.69	0.504^NS^	0.80	0.688^NS^
Hypertension	0.84	0.432^NS^	0.79	0.302^NS^	0.87	0.522^NS^	0.75	0.198^NS^
Belo Horizonte (Capital of the State)	1.33	0.256^NS^	1.31	0.330^NS^	1.57	0.084*	1.68	0.044**
Completed middle school and incomplete high school (*ref. Less than completed middle school*)	0.91	0.718^NS^	0.95	0.870NS	0.90	0.692^NS^	0.79	0.384^NS^
Completed high school	0.57	0.025**	0.59	0.050**	0.59	0.041**	0.57	0.026**
Over high school	0.40	0.013**	0.41	0.021**	0.50	0.060*	0.45	0.032**
Constant	5.34	0.001***	4.72	0.004***	4.42	0.004***	6.00	0.001***
Number of observations	1,254		1,175		1,251		1,231	
Adjusted R^2^	0.24		0.23		0.26		0.25	
Log ofLikelihood	-568.54		-528.80		-544.60		-552.62	

*Note*: ***Statistically significant at 1% margin of error (Sig. level = 99%); **Statistically significant at 5% margin of error (Sig. level = 95%); *Statistically significant at 10% margin of error (Sig. level = 90%) NS not significant

One of the main important outcomes for adequate antenatal care is birthweight. In this survey, around 12% of the mothers had a child with low birthweight, while in Brazil, this percentage was 8% in 2010 and among OECD countries, 6.6% [43, 44]. Table 6 shows the results for the logistic model estimated for the probability of having a low birthweight infant controlling for mother’s and prenatal care characteristics. The most important explanatory factors are mother’s age, multiple births and having received antenatal care by FHS. Mothers aged over 40 years old were three times more likely to have a low birthweight infant while multiple births increased the chance by 7 times (Table 6). On the other hand, women whose antenatal care was followed by FHS had a lower chance (40%) of low birthweight compared to those who received prenatal care by other type of providers (Table 6). Regarding socioeconomic condition, mother’s economic class and educational level were not important to explain the prevalence of low birthweight.

**Table 6:** Logistic regression for low birthweight, Minas Gerais, 2012

Variable	Odds ratio	*P*-values
Class C (*ref. Class A and B*)	1.85	0.024
Class D-E	1.10	0.797
North Region (*ref. Central*)	1.22	0.575
South Region	1.23	0.563
Mineiro Triangle Region	1.09	0.844
Zona da Mata Region	1.95	0.036
East Region	0.58	0.260
Belo Horizonte (Capital of the State)	4.44	<0.001
20 to 24 (*ref. 10 to 19 years old*)	2.04	0.163
25 to 29	1.29	0.629
30 to 39	1.92	0.203^NS^
Over 40 years old	3.35	0.053
Completed middle school and incomplete high school (*ref. Less than completed middle school*)	0.73	0.225^NS^
Completed high school	0.41	0.002***
Over high school	0.91	0.838^NS^
Private health insurance	1.17	0.508^NS^
Mother with diabetes	0.50	0.454^NS^
Mother with hypertension	0.45	0.013**
Multiple births	7.72	0.004***
Late prenatal care	0.58	0.150^NS^
FHS prenatal care	0.61	0.030**
Constant	0.08	0.000***
Number of observations	1,229	
Adjusted R^2^	0.10	
Log of Likelihood	-405.07	

*Note*: ***Statistically significant at 1% margin of error (Sig. level = 99%); **Statistically significant at 5% margin of error (Sig. level = 95%); *Statistically significant at 10% margin of error (Sig. level = 90%) NS not significant

## Discussion

Equity can be understood as the absence of systematic and potentially avoidable differences in healthcare access among population groups [45]. Despite its importance, based-population studies dedicated to analyze characteristics of inequalities are scarce [46]. Considering this gap, the present paper investigated inequalities in antenatal care coverage and adequacy in a context of a community based program organized in geographical areas. In Brazil, FHS is the most important national health policy to provide PHC in the public health sector. The main findings of this paper showed that the coverage of prenatal care is almost universal in catchment urban areas of FHT in Minas Gerais state including both antenatal visits and diagnostic procedures. Due to this high level of coverage, socioeconomic inequalities were not observed. These results are in accordance to the evidence found for low and middle income countries. For instance, Neal et al. [47] compared antenatal care among 35 countries with different levels of coverage and showed that societies with low coverage are more prone to exhibit inequalities.

The universal coverage observed in Minas Gerais is largely explained by the FHS that was responsible for supplying care for around 70% of the women investigated. This percentage is even higher for those without private health insurance and belonging to lower socioeconomic class, 80% and 89% respectively. Women belonging to lower socioeconomic class were at least five times more likely to receive antenatal visits and any of the antenatal tests by the FHS compared to those belonging to the highest classes. These results are strong evidence that FHS promotes equity in antenatal care in Minas Gerais. The role of FHS in the provision of antenatal care was also noticed for other localities in Brazil. Bernardes et al. [33] showed that FHS was effective in reducing prenatal care inadequacy. The authors compared prenatal care utilization in São Luis, Maranhão (Northeast region), before (1997/1998) and after FHS implementation (2010). Also Cesar et al. [48] analyzed 23 prenatal care indicators for women in Rio Grande do Sul and showed that the coverage for women receiving care under FHS was similar to the ones in private facilities. Additionally to coverage, adequacy of prenatal care was also observed since 92% of pregnant women had more than six visits and only 10% had late prenatal care. These figures are similar for women who received care by FHS suggesting that all essential components of antenatal care are supplied by the public system.

One important neonatal risk factor usually associated with prenatal care is low birthweight, which is also a predictor of child health status. Low birthweight infants are twenty times more likely to die than normal weight ones [40]. In our survey, even though average birthweight was around 3.130 kg, almost 12% of the children had low birthweight. Besides antenatal care, mother’s conditions such as nutritional status, socioeconomic level, presence of hypertension and/or diabetes, drug and tobacco addiction, and age are important risk factors for low birthweight [49–51]. In Minas Gerais, low birthweight seemed to be associated with the mother’s age and multiple pregnancy. Moreover, FHS was effective in reducing low birthweight. Women who had prenatal care through FHS were 40% less likely to have a child with low birthweight. As the coverage of antenatal care is almost universal, there were no significant differences among socioeconomic conditions highlighting once more the importance of FHS.

The success of FHS in providing adequate antenatal care is mainly explained by its design. First, as FHS is part of the public health system all services are provided without out of pocket payments, reducing the financial barriers to healthcare accessibility. Second, FHS is geographically based which guarantees proximity to the services thereby avoiding transportation barriers and minimizing opportunity costs. Usually the burden of transportation costs is higher for poorest population that lives outside the central areas. Third, CHA promotes active search of target populations including pregnant women. This strategy allows for a reduction in educational barriers since less educated individuals are less likely to realize the importance of care and where they should search for care. Fourth FHS is a community based program that helps strengthen the relationship between providers and patients. It is worth to mention that CHA are recruited to work in the communities they reside in, which assures that they can easily identify the local needs of the population. Lassi et al. [52] showed that community based interventions are effective to provide antenatal care in low income countries.

It has to be acknowledged that our data do not consider maternal, infant and child mortality since we only investigated women who had a child under five years old alive in the household at the moment of the interview. In order to take into account these outcome variables a larger sample size, not feasible to our budget, would be required. This limitation could bias our results since maternal and infant mortality can be prevented by adequate antenatal care. Consequently, our results could have underestimated the importance of FHS in promoting comprehensive equity in antenatal care since the study did not analyze data on the role of FHS in lowering infant mortality, which empirical evidence indicates that FHS is capable of reducing infant mortality rates in Brazil [26].

## Conclusions

This paper contributes to the discussion on equity in prenatal care in two ways. First, it analyzed the role of FHS in providing antenatal care considering households located in catchment areas of FHT using a representative sample of urban areas of the state of Minas Gerais. According to the 2010 Brazilian Population Census, 85% of the population in Minas Gerais lived in urban areas. This survey design helped the study to analyze the effectiveness of FHS taking into account the population that should be covered by the program. ‘The study showed that FHS contributed to the promotion of’ maternal and child care and with reduction in socioeconomic inequalities in Minas Gerais. Second, this study analyzed the role of FHS in the context of large socioeconomic disparities since Minas Gerais is marked by huge inequalities among regions and among individuals. With in all,, FHS was found to be the most important source of access among the poorest individuals without private health insurance while inequalities in prenatal care were not observed.

Equity is usually a measure of outcome concerning PHC. Guaranteed access to antenatal care services without differences regarding socioeconomic status is an important characteristic of health systems organized according the principle of universality. Besides that, there is a lack of studies producing evidences of equity, especially adopting a population-based design [53]. Considering this scenario the contributions provided by our work highlight the importance of strengthen the public primary health care, once it can contribute do overcome access barriers and increase the provision of preventive services.

## Additional file

Additional file 1. Distribution of pregnant women who underwent prenatal tests (total and by FHS) according to economic class (%) - Minas Gerais - 2012. (DOCX 17 kb)

## Abbreviations

CHA: Community health agents
FHS: Family health strategy
FHT: Family health teams
PHC: Primary health care
STD: Sexually transmitted disease
