# Supplementary material for: Family health strategy and equity in prenatal care: a population based cross-sectional study in Minas Gerais, Brazil
Source: Int J Equity Health. 2017 Jan 21;16:24. doi: 10.1186/s12939-016-0503-9 (PMC5251278; doi:10.1186/s12939-016-0503-9)
Supplement: Additional file 1. — Distribution of pregnant women who underwent prenatal tests (total and by FHS) according to economic class (%) - Minas Gerais - 2012. (DOCX 17 kb) [file 12939_2016_503_MOESM1_ESM.docx]

**TABLE S1**

**Distribution of pregnant women who underwent prenatal tests (total and by FHS) according to economic class (%) - Minas Gerais - 2012**

| **Economic class** | **Blood test** | | **Toxoplasmosis test** | | **Urine test** | | **STD test** | |
| --- | --- | --- | --- | --- | --- | --- | --- | --- |
|  | **%** | | **%** | | **%** | | % | |
|  | **Total (N=1,406)** | **By FHS (N=744)** | **Total (N=1,221)** | **By FHS (N=655)** | **Total (N=1,403)** | **By FHS (N=762)** | **Total (N=1,377)** | **By FHS (N=715)** |
| A-B | 100.00 | 38.33 | 90.61 | 35.37 | 100.00 | 39.51 | 99.55 | 38.34 |
| C | 98.58 | 53.68 | 85.95 | 48.87 | 98.44 | 54.78 | 96.39 | 51.09 |
| D-E | 98.99 | 64.75 | 89.85 | 65.80 | 98.33 | 67.05 | 97.13 | 66.06 |
| **Total** | **99.01** | **52.79** | **87.96** | **49.88** | **98.77** | **54.19** | **97.29** | **51.67** |
